# Bidirectional Terahertz Vortex Beam Regulator

**DOI:** 10.3390/ma15238639

**Published:** 2022-12-03

**Authors:** Jiusheng Li, Fenglei Guo, Shuping Zhang, Chao Liu

**Affiliations:** 1 Centre for THz Research, China Jiliang University, Hangzhou 310018, China; 2 Information Engineering Department, Xinjiang Institute of Technology, Aksu 735400, China

**Keywords:** terahertz technology, terahertz vortex beam regulator, multilayer metasurface

## Abstract

Most of the reported vortex beam generators with orbital angular momentum (OAM) in the terahertz region only operate in either the reflection mode or the transmission mode, which greatly limits the integration and application in terahertz technology systems. Herein, we propose a full-space vortex beam regulator at two different frequencies. By changing the VO_2_ phase transition state, the transmission and reflection mode OAM beams can be flexibly controlled by a single metasurface. For the transmission mode, the proposed structure realizes an OAM beam at the topological charges of *l* = 1 and 2 at 0.6 THz and 1.4 THz. For the reflection mode, our structure generates an OAM beam at the topological charges of *l* = 1 and 2 at 0.9 THz and 1.5 THz. Based on the superposition theorem and convolution operation principle, the regulation of an OAM vortex beam with a specific deflection angle and a symmetrical deflection OAM vortex beam are realized. The designed metasurface integrates multiple transmitted and reflected vortex beam functions in full space and has potential application in different terahertz systems.

## 1. Introduction

A vortex beam generator with OAM has a phase factor of exp(i*lφ*) (Here, *φ* denotes the azimuth angle, and *l* represents an integer topological charge value), which can theoretically increase the information capacity infinitely, and the OAM vortex beams with different integer orders are usually orthogonal to each other [1,2]. Recently, some progress has been made in the investigation using the metasurface to generate an OAM beam. For example, Yu et al. proposed a V-shaped metasurface structure to produce OAM beams in optical regions [3]. Zhang et al. used a strip-line metasurface to realize different polarization vortex beams by using the Pancharatnam–Berry principle [4]. Rouhi et al. [5] demonstrated a hybrid metasurface to generate the conversion of vortex beams. Wang et al. [6] used a cascade structure to generate a transmissive Bessel OAM beam within a broad frequency band. Xie et al. [7] employed Pancharatnam–Berry metasurfaces to generate different OAM-mode vortex beams. In reference [8], a grating structure was used to generate a broadband-transmitted vortex beam from 8 GHz to 13 GHz. Fan et al. [9] presented a trilayered metasurface structure to generate *l* = 1 and *l* = 2 OAM beams. These metasurface structures can be roughly divided into reflection-mode OAM and transmission-mode OAM, which utilizes only half the electromagnetic space. In order to make full use of the electromagnetic space resources, it is of great significance to manipulate both transmissive and reflective electromagnetic waves in a metasurface. More recently, there have also been some reports on the whole-space metasurfaces. Wang et al. [10] realized the regulation of the electromagnetic wave in a whole space under the incidence of a linearly polarized (LP) wave. The vortex generators designed in reference [11,12,13] worked in the transmission and reflection modes generating multifunctional beams. Xin et al. [14] used a 2-bit bilayer-encoded metasurface to achieve vortex beams under LP wave incidence at the Ku and Ka communication bands. In 2020, Dong et al. [15] demonstrated a metal–VO_2_ hybrid metasurface to achieve a switchable broadband asymmetric transmission in the terahertz region. In 2021, Li et al. [16] designed a single-layer metasurface to generate an arbitrary value of a terahertz vortex beam, at the same time, the beam showed stable propagation characteristics and kept a high fidelity over a broad frequency range. However, the reported metasurface-based OAM beam generators only realized the regulation of a single-frequency band and single transmission direction. Therefore, exploiting a terahertz vortex regulator with bidirectional and dual-frequencies is of great significance.

In this article, we design a dual-band, full-space terahertz vortex beam regulator. The meta-element consists of four layers from top to bottom: single-open metal outer ring and middle notched inner ring hybrid structure, polyimide, vanadium dioxide film, single-open metal outer ring, and middle notched inner ring hybrid structure. By controlling the phase transition state of VO_2_, our metasurface realized both the transmission and reflection modes of terahertz OAM beams at different frequencies. Our work has good application prospects in terahertz communication and imaging systems.

## 2. Metal Particle

**Figure 1:**
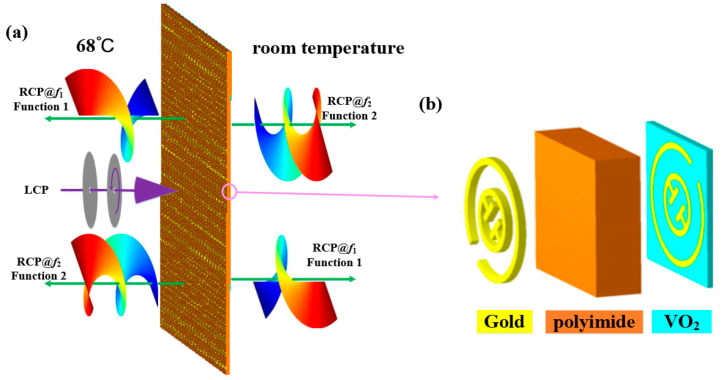
Schematic diagram of the proposed bidirectional OAM generator at two frequencies (**a**) and the perspective view of the meta-element (**b**).

**Figure 2:**
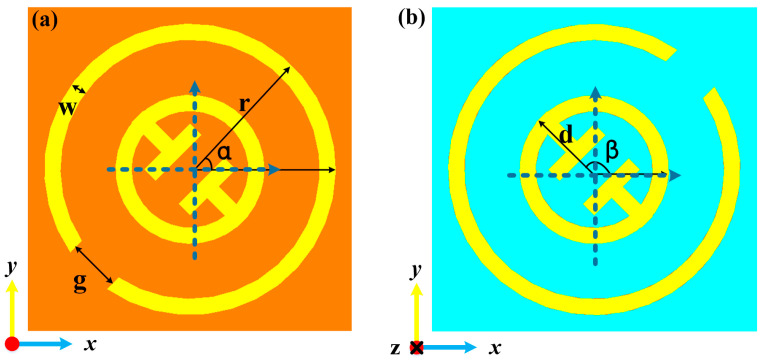
Top (**a**) and back (**b**) views of the proposed meta-element structure.

Figure 1 illustrates the schematic of the proposed bidirectional terahertz vortex regulator, which is made of multilayer meta-element array patterns. The top layer pattern is composed of a single-open metal outer ring and middle notched inner ring hybrid structure, and the bottom layer is the same pattern as the top layer with 90° rotation (Herein, from left to right, the multilayer meta-element is named as top layer, substrate layer, and bottom layer). The CST Studio Suite 2018 simulation software was employed to optimize the meta-elements geometric dimensions [17]. The metal thickness was 0.2 μm, and the period of the meta-element was set to be 100 μm. The thickness of the intermediate polyimide layer was 30μm. The optimized geometry parameter values of the meta-elements were as follows: w = 5 μm, g = 16 μm, r= 45 μm, and d = 23 μm. The orientations of the top and bottom single-open metal outer ring and middle notched inner ring hybrid metasurface were set as *α* and *β* with respect to the *x*-axis, respectively. Polyimide film with a relative permittivity of ε_r_ = 3.5 × (1 + 0.0027i) was selected as the dielectric substrate. The material of the metal ring was gold, and its conductivity is 4.56 × 10^7^ S/m. Under the left-handed circularly polarized (LCP) wave irradiation, the vortex beams with various topological charges were achieved at two frequencies. The transmissive mode and reflective mode of the terahertz wave can be controlled by manipulating the phase change state of vanadium dioxide (VO_2_). Figure 2a,b give the top and back views of the meta-element of our metasurface.

The dielectric constant of VO_2_ in the terahertz band can be given by the Drude model

(1) $$\varepsilon (\omega )=\varepsilon_{\infty }-\frac{\sigma }{\sigma_{0}}\frac{\omega_{p}^{2}(\sigma_{0})}{\omega^{2}+i\gamma \omega }$$

where *ε*_∞_ = 12; *ω_p_*(*σ*) is the plasma frequency, *σ*_0_ = 3 × 10^5^ Ω^−1^cm^−1^ and *ω_p_*(*σ*_0_) = 1.40 × 10^15^ rad/s; and *γ* is the collision frequency, *γ* = 5.75 × 10^13^ rad/s. Here, we presumed the conductivity of VO_2_ in the metallic state and the insulated state (2 × 10^5^ S/m and 200 S/m), respectively. According to the Pancharatnam–Berry (P–B) phase principle, when a plane wave beam is normally illuminated on the designed metasurface, the transmission matrix T of the transmissive and incident field can be written by

(2) $$T_{CP}=\left(\begin{array}{cc} T_{LL} & T_{LR}\\ T_{RL} & T_{RR} \end{array}\right)=\left(\begin{array}{cc} \frac{t_{xx}+t_{yy}+i(t_{xy}-t_{yx})}{2} & \frac{t_{xx}-t_{yy}-i(t_{xy}+t_{yx})}{2}\\ \frac{t_{xx}-t_{yy}+i(t_{xy}+t_{yx})}{2} & \frac{t_{xx}+t_{yy}-i(t_{xy}-t_{yx})}{2} \end{array}\right)$$

In the formula, *t*_xx_, *t*_xy_, *t*_yx_ and *t*_yy_ represent the transmissive coefficients of the LP wave, and the first subscript describes the transmitted wave polarization state, while the second subscript represents the incident wave polarization state. The electromagnetic wave beam propagation direction after passing through the meta-element can be expressed by using a rotation matrix with the rotation angle of *θ*

(3) $$R(\theta )=\left(\begin{array}{cc} \cos\theta & \sin\theta \\ -\sin\theta & \cos\theta \end{array}\right)$$

In Formula (2), the transmission matrix can be described

(4) $$\begin{array}{l} T_{CP}^{*}=R(-\theta )T_{CP}R(\theta )=\\ \left[\begin{array}{cc} \frac{t_{xx}+t_{yy}-(t_{xx}-t_{yy})\sin2\theta +i(t_{xy}-t_{yx})\cos2\theta }{2} & \frac{(t_{xx}-t_{yy})\cos2\theta -i(t_{xy}+t_{yx})+i(t_{xy}-t_{yx})\sin2\theta }{2}\\ \frac{(t_{xx}-t_{yy})\cos2\theta +i(t_{xy}+t_{yx})+i(t_{xy}-t_{yx})\sin2\theta }{2} & \frac{t_{xx}+t_{yy}+(t_{xx}-t_{yy})\sin2\theta -i(t_{xy}-t_{yx})\cos2\theta }{2} \end{array}\right] \end{array}$$

The incident waves can be given by

(5) $$E_{i}=E_{0}{\left(1\pm i\right)}^{T}$$

where “+” represents the LCP wave normal incidence, and “−” is the RCP wave normal incidence. The transmission field of our structure can be written by

(6) $$E_{\mathrm{out}}^{t}=T_{CP}^{*}\times E_{\mathrm{in}}=\frac{1}{2}(t_{xx}+t_{yy}+t_{xy}+t_{yx})\left(\begin{array}{c} 1\\ i \end{array}\right)+\frac{1}{2}(t_{xx}-t_{yy}+t_{xy}-t_{yx})e^{i2\theta }\left(\begin{array}{c} i\\ 1 \end{array}\right)$$

Similarly, the reflection field of the proposed metasurface can be given by

(7) $$E_{\mathrm{out}}^{r}=\frac{1}{2}(r_{xx}+r_{yy}+r_{xy}+r_{yx})\left(\begin{array}{c} 1\\ i \end{array}\right)+\frac{1}{2}(r_{xx}-r_{yy}+r_{xy}-r_{yx})e^{i2\theta }\left(\begin{array}{c} i\\ 1 \end{array}\right)$$

It is worth noting from the above formula that the first term of the outgoing wave is the copolarized wave without introducing the phase factor, and the second term is the additional phase CP wave.

**Figure 3:**
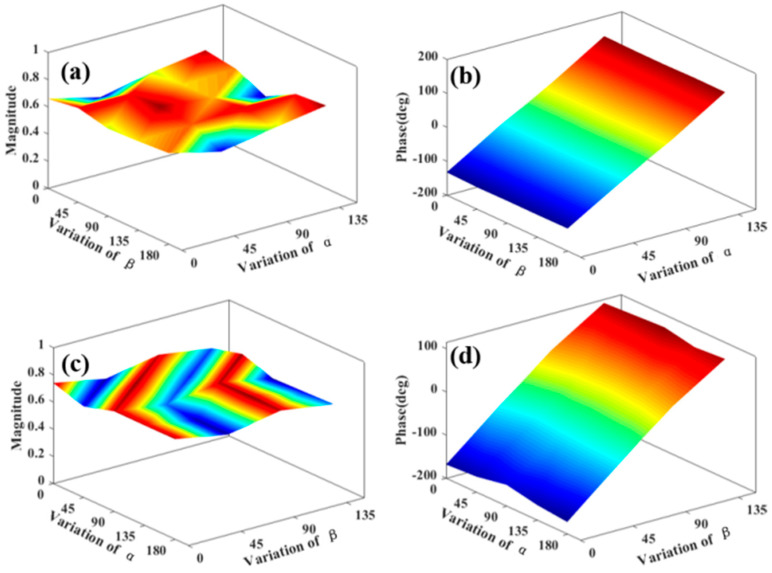
Transmissive mode amplitude and phase of our metasurfaces at room temperature. (**a**,**b**) at 0.6 THz and (**c**,**d**) 1.4 THz.

**Figure 4:**
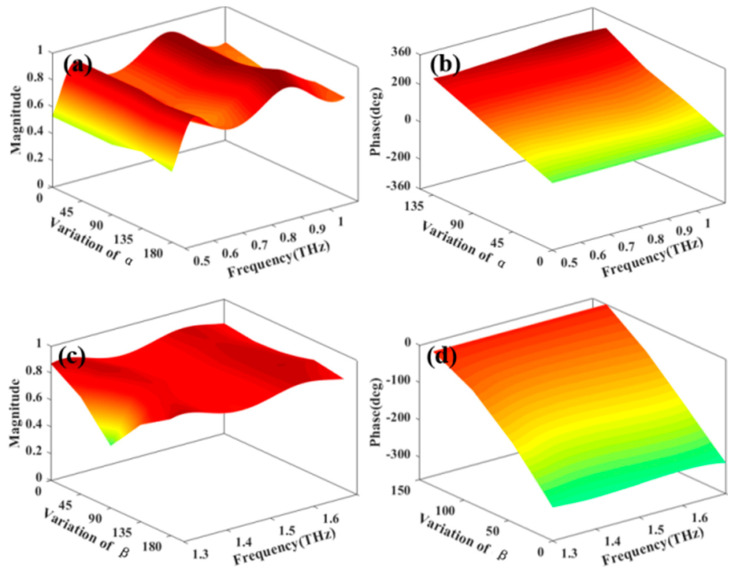
Reflective mode amplitude and phase of our metasurface at 68 °C. (**a**,**b**) at 0.9 THz and (**c**,**d**) at 1.5 THz.

As VO_2_ varies from dielectric state to metallic state, its conductivity increases sharply. By using the VO_2_ phase transition, we can achieve a free switching between the transmissive and reflective modes of the terahertz waves. Figure 3 illustrates the transmissive mode amplitude and phase of the metasurface structure at 0.6 THz and 1.4 THz under room temperature. From the figure, one can see that the phase change of the meta-element is twice that of the metal outer ring angle α, and the phase change is independent of the metal inner ring angle β at 0.6 THz. For 1.4 THz, the meta-element phase change is twice that of the metal inner ring angle β, while the phase change is independent of the metal outer ring angle α. Similarly, Figure 4 displays the reflective mode amplitude and phase of our structure at 0.9 THz and 1.5 THz under 68℃. At 0.9 THz, the phase change of the meta-element is twice that of the metal outer ring angle α, and the phase change is independent of the metal inner ring angle β. For 1.5 THz, the meta-element phase change is twice that of the metal inner ring angle β, while the phase change is independent of the metal outer ring angle α. The results show that the phase of our structure can be independently adjusted at different frequencies.

## 3. Simulation and Analysis

### 3.1. Normal Transmission Vortex Generator

**Figure 5:**
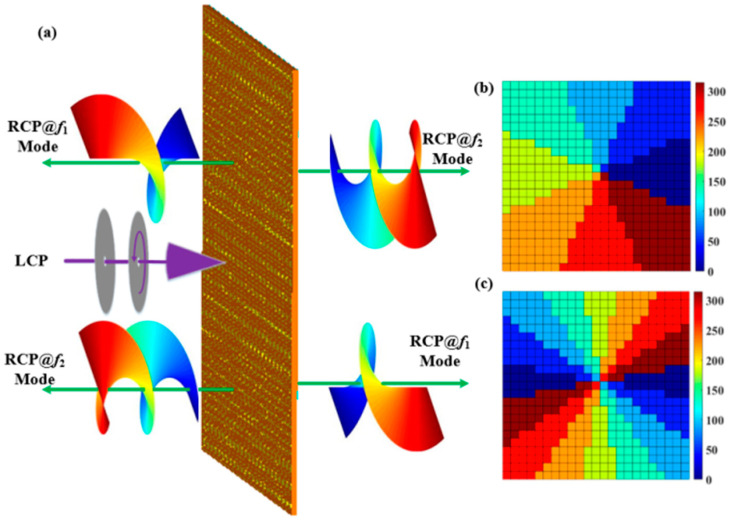
(**a**) Function and perspective view of the proposed axial transmission vortex beam generator, (**b**) phase distribution of metal outer ring, and (**c**) phase distribution of metal inner ring.

The axial transmission vortex generators with topological charges of 1 and 2 were arranged by using the designed meta-elements, as displayed in Figure 5. In this case, the phase of each position (*x*,*y*) in the proposed metasurface needs to meet the following relation:

(8) $$\varphi_{1}(x,y)=l\cdot \tan^{-1}\left(\frac{y}{x}\right)$$

In the formula, *l* denotes the number of topological charges. According to the P–B phase principle, the rotation angles (*α*) and (*β*) of the metal ring are required to generate vortex beams with topological charges of 1 and 2. Figure 5 depicts two full-space crosspolarized OAM beams (Here, *l* = 1 and *l* = 2.) by adjusting the phase transition state of the vanadium dioxide under the incident circularly polarized (CP) terahertz wave at different frequencies.

The mode purity is calculated by the following formula:

(9) $$\left\{\begin{array}{c} \alpha (\varphi )=\sum \begin{array}{c} +\infty \\ l=-\infty \end{array}A_{l}\cdot \exp(il\varphi )\\ A_{l}=\frac{1}{2\pi }\int \begin{array}{c} \pi \\ -\pi \end{array}d\varphi \alpha (\varphi )\cdot \exp(il\varphi ) \end{array}\right.$$

where α (φ) is the phase sampling, exp (ilφ) It is a spiral harmonic. Here, the purity of the mode generating the vortex beam is defined as the ratio of the main mode power to the total power of all modes.

**Figure 6:**
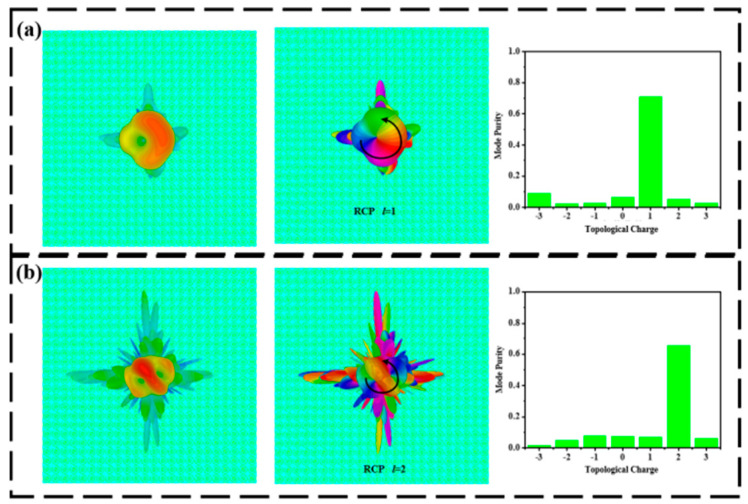
Far-field intensity distribution, phase, and mode purity of axially transmissive metasurfaces at room temperature. (**a**) 0.6 THz and (**b**) 1.4 THz.

**Figure 7:**
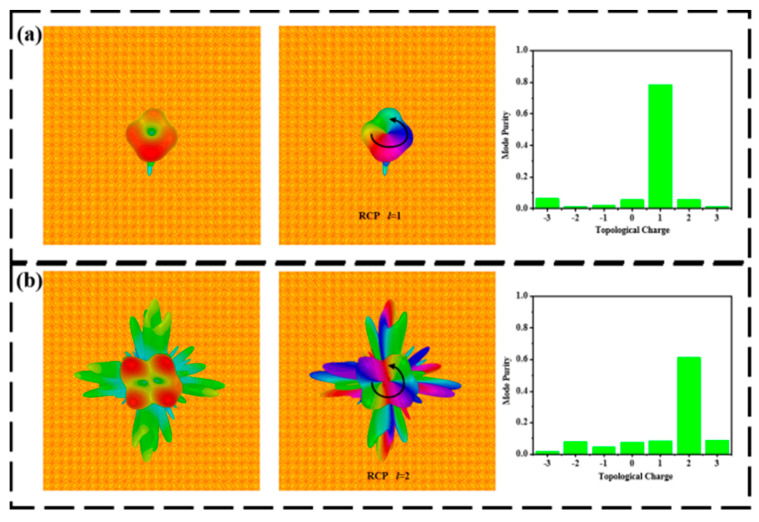
Far-field intensity distribution, phase, and mode purity of axially reflective metasurfaces at 68 °C. (**a**) 0.9 THz and (**b**) 1.5 THz.

Figure 6a illustrates the transmitted far-field intensity, phase, and mode purity of the LCP wave normal incidence at the frequency of 0.6 THz under room temperature. The designed metasurface generates a vortex beam with a mode purity of 70.8% at a topological charge of 1. At the frequency of 1.4 THz, the far-field, phase distribution, and mode purity of the transmitted terahertz waves are shown in Figure 6b. Noting that the proposed metasurface generates a vortex beam with a mode purity of 65.5% at a topological charge of 2, Figure 7a shows the reflected far-field, phase distribution, and mode purity of the right-handed circularly polarized (RCP) wave normal incidence at the frequency of 0.9 THz under 68 °C. The mode purity of the generated vortex beam is 78. 4% at a topological charge of 1. For 1.5 THz, the far-field, distribution, and mode purity of the reflective terahertz wave are presented in Figure 7b. The mode purity of the vortex beam is 62.2% at a topological charge of 2.

### 3.2. Inclined Transmission Vortex Generator

When the OAM phase and the gradient phase are superimposed, the vortex beam will carry the deflection angle along +*x* direction, and its phase distribution can be calculated by

(10) $$\varphi_{2}(x,y)=\varphi_{1}(x,y)+\left(2\pi \cdot \sin\theta \right)/\lambda$$

**Figure 8:**
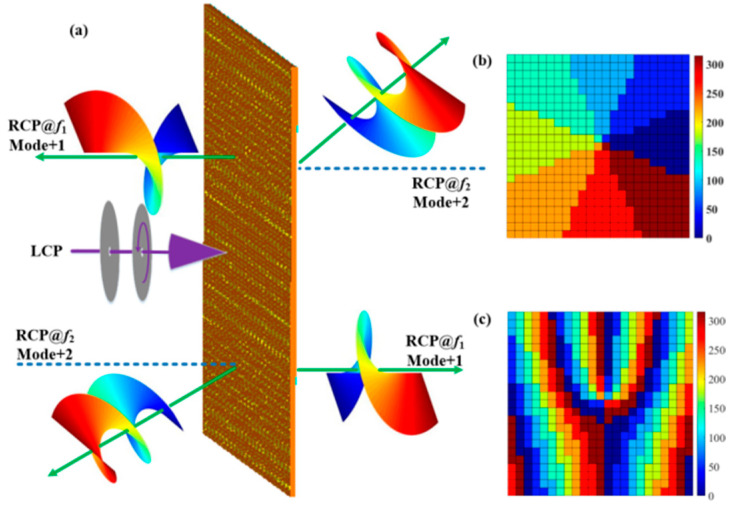
(**a**) Function and perspective view of the transmission deflective terahertz OAM generator, (**b**) phase distribution of metal outer ring, and (**c**) phase distribution of metal inner ring.

Here, *θ* represents the deflection angle, which can be given by the following formula:

(11) $$\theta =\sin^{-1}\left(\frac{\lambda }{\tau }\right)$$

where *τ* denotes the period of the coding sequence. Herein, *τ* = 8P = 800 μm. The calculated deflection angles are 15.5° at 1.4 THz and 14.4° at 1.5 THz. Figure 8a gives the deflected transmission and reflection modes of the OAM beam at different frequencies by adjusting the phase transition state of the VO_2_ film under CP wave incidence. Figure 8b,c plot the phase distributions of the metal inner and outer rings by using the superposition theorem.

**Figure 9:**
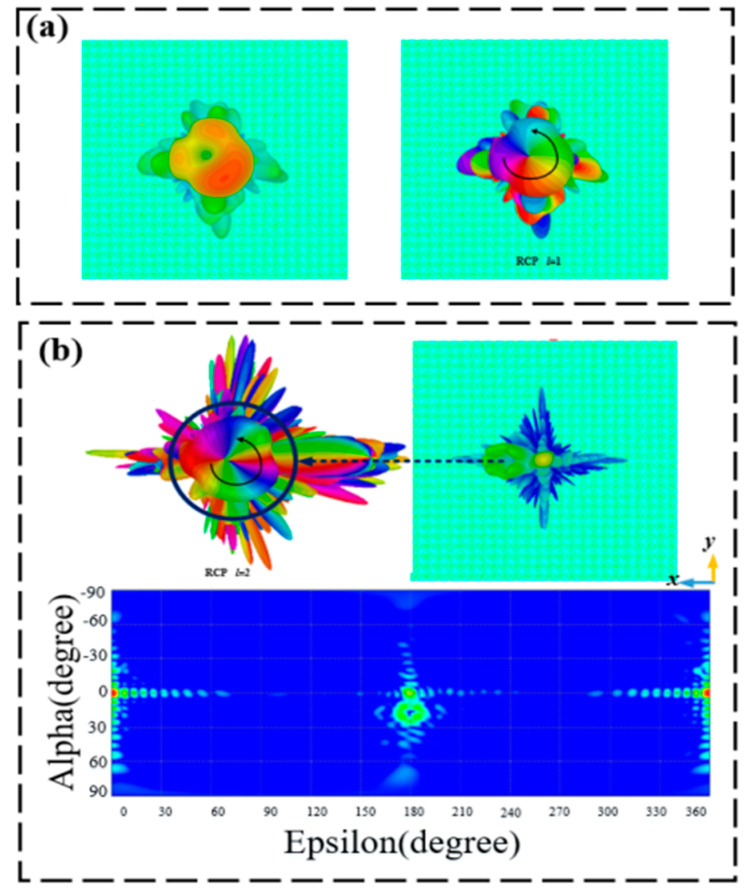
Three-dimensional far-field, phase distribution, and two-dimensional electric field diagrams of the transmission deflective OAM at room temperature. (**a**) 0.6 THz and (**b**) 1.4 THz.

**Figure 10:**
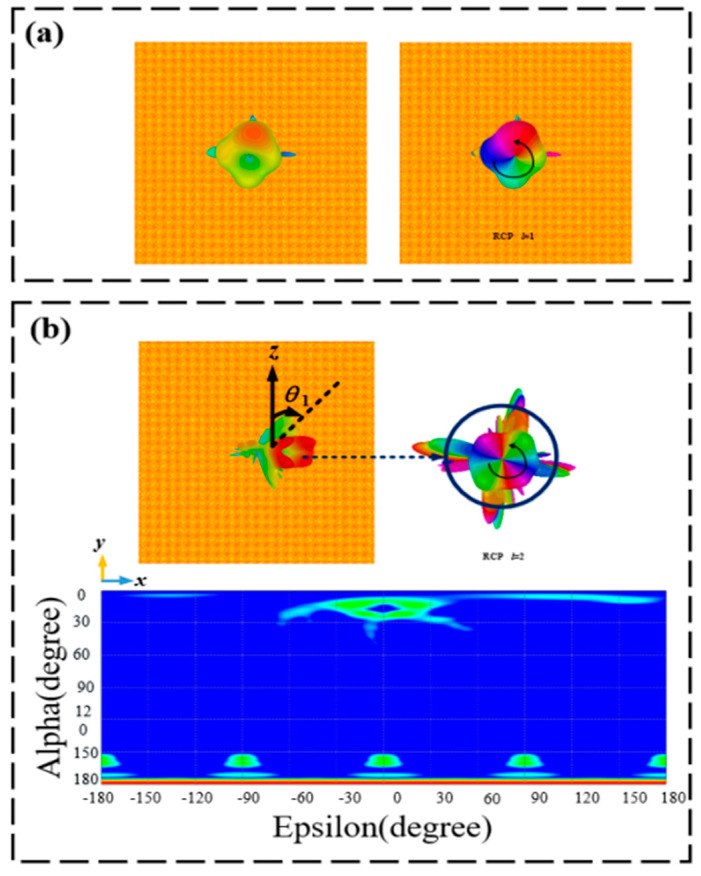
Three-dimensional far-field intensity, phase, and two-dimensional electric field diagrams of the reflected deflection OAM at 68 °C. (**a**) 0.9 THz and (**b**) 1.5 THz.

Figure 9a shows the three-dimensional far-field intensity, phase distribution, and two-dimensional electric field diagram of the transmitted OAM vortex beam under LCP wave normal incidence at the frequency of 0.6 THz at 25 °C. One can see that the vortex beam displays a topological charge of *l* = 1. As for 1.4 THz, Figure 9b displays the three-dimensional, far-field intensity, phase distribution, and two-dimensional electric field diagram of the transmitted OAM vortex beam at room temperature. From the figure, we find that the structure behaves like vortex beams having a deflection angle of 15.5° and a topological charge of 2. Likewise, for operating at 68 °C, Figure 10a shows the three-dimensional far-field intensity, phase distribution, and two-dimensional electric field diagram of the reflected OAM vortex beam under LCP wave normal incidence at 0.9 THz. The proposed structure achieves a vortex beam with a topological charge of 1. At the frequency of 1.5 THz, the generated vortex beam shows a topological charge of 2 and a deflection angle of 14.4°. The three-dimensional far-field intensity, phase diagram, and two-dimensional electric field of the reflected OAM vortex beam are shown in Figure 10b.

### 3.3. Beam-Splitting Transmission Terahertz Vortex Generator

**Figure 11:**
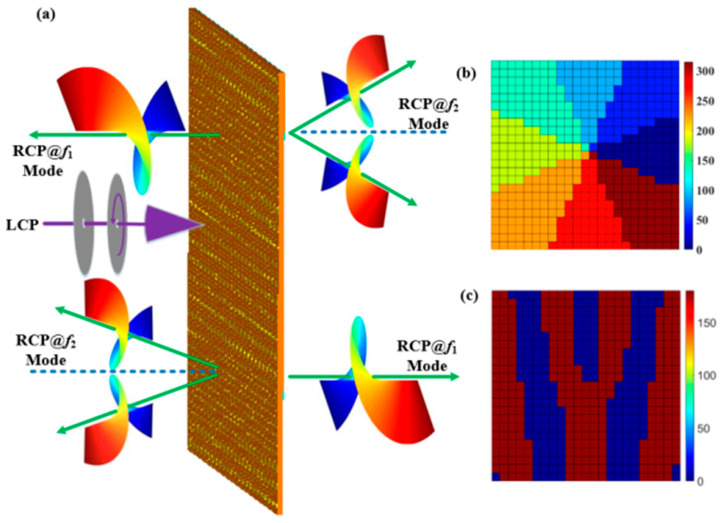
(**a**) Function schematic diagram of the proposed beam-splitting terahertz vortex generator, (**b**) Phase distribution of metal outer ring, and (**c**) Phase distribution of metal inner ring.

A beam-splitting transmission OAM generator can be realized by convoluting the metasurface (i.e., Figure 1) with the beam-splitting phase. The proposed structure produces two OAM beams with different topological charges of *l* = ±1. The phase after convolution operation of the two OAM beams can be expressed by [18]

(12) $${f(x}_{\lambda })\cdot e^{{\mathrm{jx}}_{\lambda }{\sin\theta }_{0}}\overset{\mathrm{FFT}}{\Leftrightarrow }{F(\sin\theta }_{0}{)*\delta (\sin\theta \;-\;\sin\theta }_{0}{)=F(\sin\theta \;-\;\sin\theta }_{0})$$

where the item e^jsinθ₀xλ^ describes the metasurface with unity-scattering amplitude and gradient phase distribution, and Figure 11a shows the bidirection OAM vortex beams at a circularly polarized wave incidence by adjusting the VO_2_ film phase transition state at two different frequencies. Figure 11b,c display the phase distributions of the metal inner and outer rings by using convolution operation.

**Figure 12:**
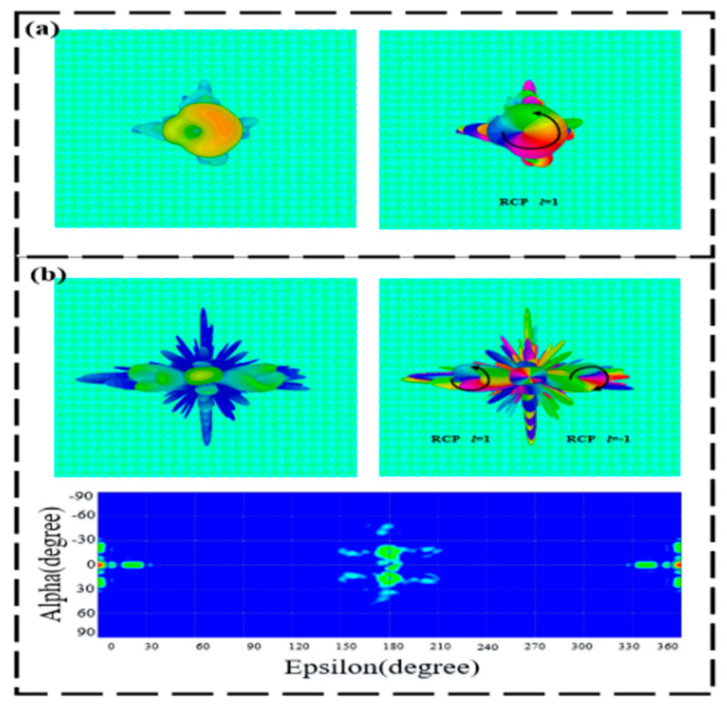
Three-dimensional, far-field, and phase diagram of the transmissive-splitting OAM generators at room temperature. (**a**) 0.6 THz and (**b**) 1.4 THz.

**Figure 13:**
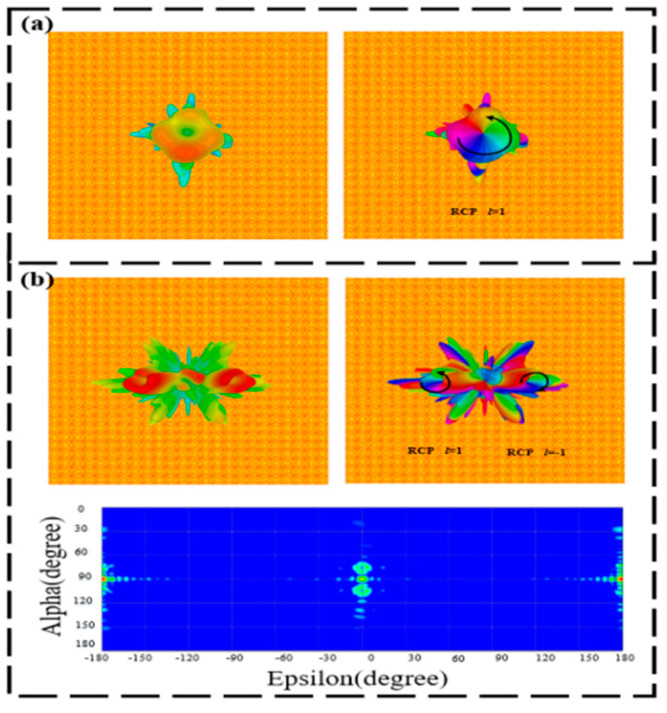
Three-dimensional, far-field, and phase diagram of the reflective-splitting OAM generators at 68 °C. (**a**) 0.9 THz and (**b**) 1.5 THz.

Figure 12a displays the far-field and phase diagram of the RCP transmission OAM vortex beam with *l* = 1 under the LCP wave incidence of 0.6 THz at room temperature. At the frequency of 1.4 THz, the LCP terahertz wave is normally incident on the metasurface to produce an RCP transmission OAM vortex beam with *l* = ±1 and a deflection angle of ±15.5°, as illustrated in Figure 12b. When the operating temperature becomes 68 °C, the LCP wave at 0.9 THz is incident normally on the designed metasurface to realize the RCP-reflective OAM beam with *l* = 1, as illustrated in Figure 13a. Similarly, the LCP wave is normally incident to our proposed structure, which generates an RCP transmission OAM beam with *l* = ±1 and a deflection angle of ±14.4°. The corresponding three-dimensional, far-field intensity, phase diagram, and two-dimensional electric field are shown in Figure 13b.

## 4. Conclusions

In this work, we propose a metasurface to produce transmissive and reflective mode vortex beams with an OAM at two different frequencies. By changing the phase transition state of the VO_2_, the transmission mode and reflection mode of the terahertz OAM vortex beams can be dynamically adjustable. In addition, using the principle of the superposition theorem and convolution operation, the OAM vortex beam with specific inclination and the symmetrical inclination OAM vortex beam are numerically demonstrated. The simulated results by using CST software are in good agreement with the theoretically calculated predictions. This study provides an effective way to integrate many functionalities into a metasurface.

## Data Availability

Data available on request from the authors.
